# Compression strength and damage model of frozen silty clay in Xing’an Baikal permafrost under temperature effects

**DOI:** 10.1038/s41598-025-06222-3

**Published:** 2025-07-01

**Authors:** Kezheng Chen, Shuai Huang, Xiujuan Li, Haiping Liu, Yang Yang, Yanjie Liu, Lin Ding

**Affiliations:** 1 College of Mechanical and Electrical Engineering, Northeast Forestry University, Harbin, 150040 People’s Republic of China; 2 Key Laboratory of Sustainable Forest Ecosystem Management (Ministry of Education), School of Ecology, Northeast Forestry University, Harbin, 150040 People’s Republic of China; 3 School of Civil Engineering, Heilongjiang University, Harbin, 150080 People’s Republic of China; 4 College of Civil and Architectural Engineering, Heilongjiang Institute of Technology, Harbin, 150050 People’s Republic of China; 5 Heilongjiang Province Hydraulic Research Institute, Harbin, 150080 People’s Republic of China

**Keywords:** Xing’an Baikal permafrost, Silty clay, Compression strength, Damage model, Civil engineering, Cryospheric science

## Abstract

The Daxing’anling, situated within the high-latitude transition zone between continuous and sporadic permafrost, mark the southern boundary of the Northern Hemisphere’s permafrost distribution. The thermally sensitive Xing’an Baikal permafrost in this region was investigated through uniaxial compression tests on remolded silty clay under controlled freezing temperatures (− 7.5 to − 0.5 °C). Results revealed a triphasic strength-temperature relationship: strength increased at 79.99 kPa/°C between − 0.5 and − 2.0 °C, surged to 1842.00 kPa/°C from − 2.0 to − 3.0 °C, then declined to 316.20 kPa/°C below − 3.0 °C. A brittle-ductile transition occurred at − 3.0 °C, shifting failure modes from plastic to brittle deformation. Building on Lemaitre’s strain equivalence principle and Weibull statistics, we developed a dual-variable damage model integrating thermal and mechanical damage, enabling quantitative cryogenic damage assessment, coupled damage evolution equations, and full temperature-regime stress–strain predictions. This work advances theoretical tools for engineering stability evaluation in the Xing’an Baikal permafrost environments.

## Introduction

The Daxing’anling is situated at the southern boundary of permafrost in the Northern Hemisphere^1^, serving as a transition zone between permafrost and seasonally frozen ground. The permafrost in this area is commonly referred to as Xing’an Baikal permafrost^2–4^. The distribution of permafrost in the Daxing’anling is influenced by various factors, including biological, climatic, geomorphological, and human activities^5^, primarily following a north-to-south pattern: continuous permafrost, continuous and island permafrost, and sparsely island permafrost^6,7^.

Based on thermal conditions, permafrost can be classified into four categories: high-temperature extremely unstable permafrost (*Tep* > − 0.5 °C), high-temperature unstable permafrost (− 1.0 °C ≤ *Tep* ≤ − 0.5 °C), low-temperature basically stable permafrost (− 2.0 °C ≤ *Tep* < − 1.0 °C), and low-temperature stable permafrost (*Tep* < − 2.0 °C)^8^. Data from the China-Russia Crude Oil Pipeline (CRCOP) and other published literature indicate that the most prevalent type of permafrost in the Daxing’anling is high-temperature unstable permafrost, followed by low-temperature basically stable permafrost. Low-temperature stable permafrost is less frequently observed; however, in extreme instances, permafrost temperatures in certain areas can reach approximately − 7.5 °C^9–12^.

In the Daxing’anling, the thickness of permafrost varies from 5 to 100 m, with occasional instances exceeding 100 m. This thicker permafrost primarily develops in marshy regions of intermountain depressions and river valley terraces where moss and peat layers exist^13^. Consequently, numerous structures in the Daxing’anling are constructed on permafrost, with their foundational load-bearing layers also situated within it^14^. The thermal conditions of the permafrost significantly influence the load-bearing capacity of these foundations^15,16^. Therefore, investigating the mechanical properties of permafrost at varying temperatures is essential for evaluating the stability of engineering foundations in permafrost regions.

The constitutive relation is a mathematical expression that defines the relationship between stress and strain in materials^17^. In geotechnical analysis, establishing a constitutive relation that accurately represents the deformation characteristics of soil is fundamental to ensuring the validity of mechanical analysis results^18,19^. Constitutive relations for frozen soils are generally categorized into three types: nonlinear elastic models, elastic–plastic models, and viscoelastic-plastic models^20^. Building on these three models, damage models that incorporate damage variables can more accurately describe the deformation behavior of frozen soils^21^. Although constitutive models of frozen soil have been extensively studied, existing models still exhibit critical limitations. For instance, most models consider only mechanical damage under loading while neglecting microstructural degradation caused by temperature fluctuations^22^, leading to significant deviations in predicting frozen soil strength attenuation. Moreover, the majority of current models fail to explicitly account for the interaction between thermal and mechanical damage, making it difficult to accurately simulate the failure behavior of frozen soil under combined thermal–mechanical loading.

This study investigates the Xing’an Baikal permafrost through uniaxial compression tests, elucidating temperature effects on frozen soil behavior (stress–strain curves, secant modulus) and strength characteristics. Building on Lemaitre’s strain equivalence principle and Weibull distribution^23,24^, we propose the first dedicated dual-damage coupling model for this permafrost type that simultaneously quantifies both thermal and mechanical damage. The model achieves continuous damage characterization across the entire temperature spectrum (from warm to cold frozen soils), providing more reliable design criteria for engineering projects in the Xing’an Baikal permafrost regions.

## Materials and methods

### Materials

**Fig. 1:**
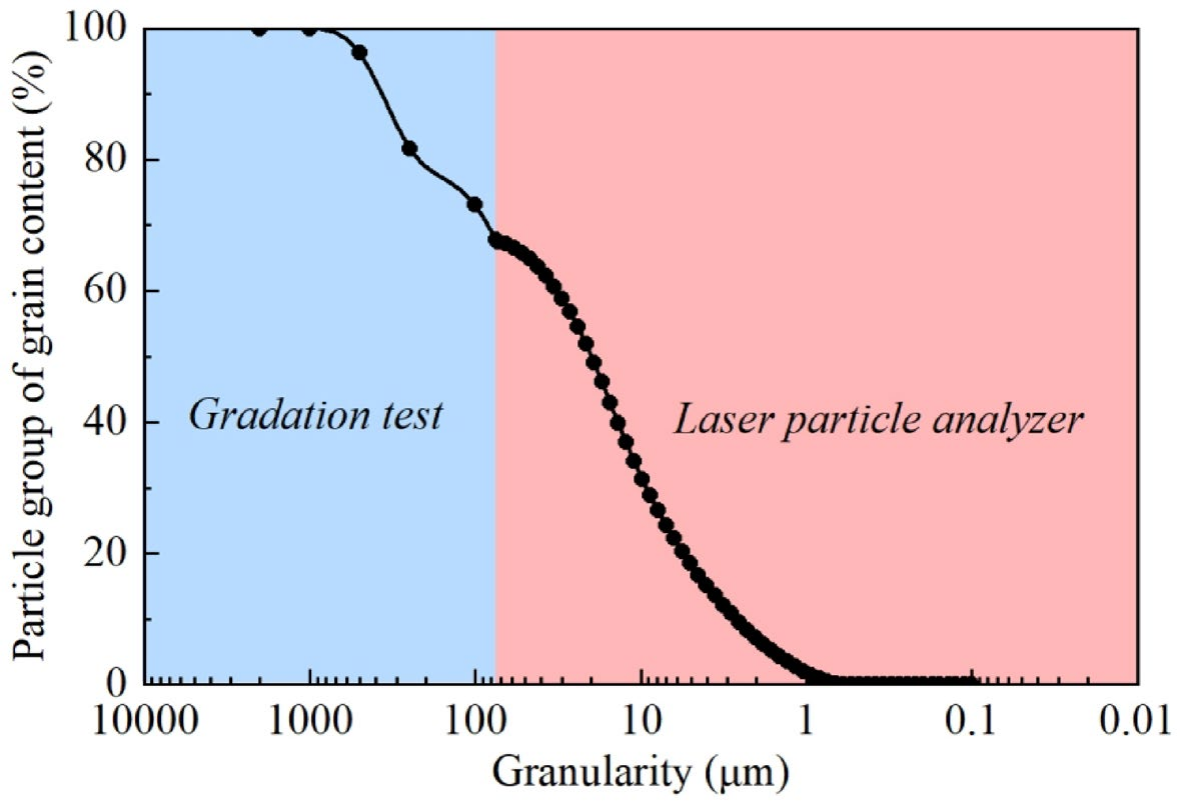
Soil particle size distribution curve.

To determine the particle size distribution characteristics, the soil sample was analyzed using both sieve and laser diffraction to obtain the particle size distribution curve and specific surface area. The oven-dried sample was first thoroughly ground, then sieved through standard test sieves. For particles smaller than 75 μm, a 4% sodium hexametaphosphate dispersing solution mixed with distilled water (1:6) was added, followed by vigorous shaking to ensure complete particle dispersion before laser diffraction analysis. The combined methods yielded the particle size distribution curve (Fig. 1) and specific surface area (390.5 m^2^/kg).

**Fig. 2:**
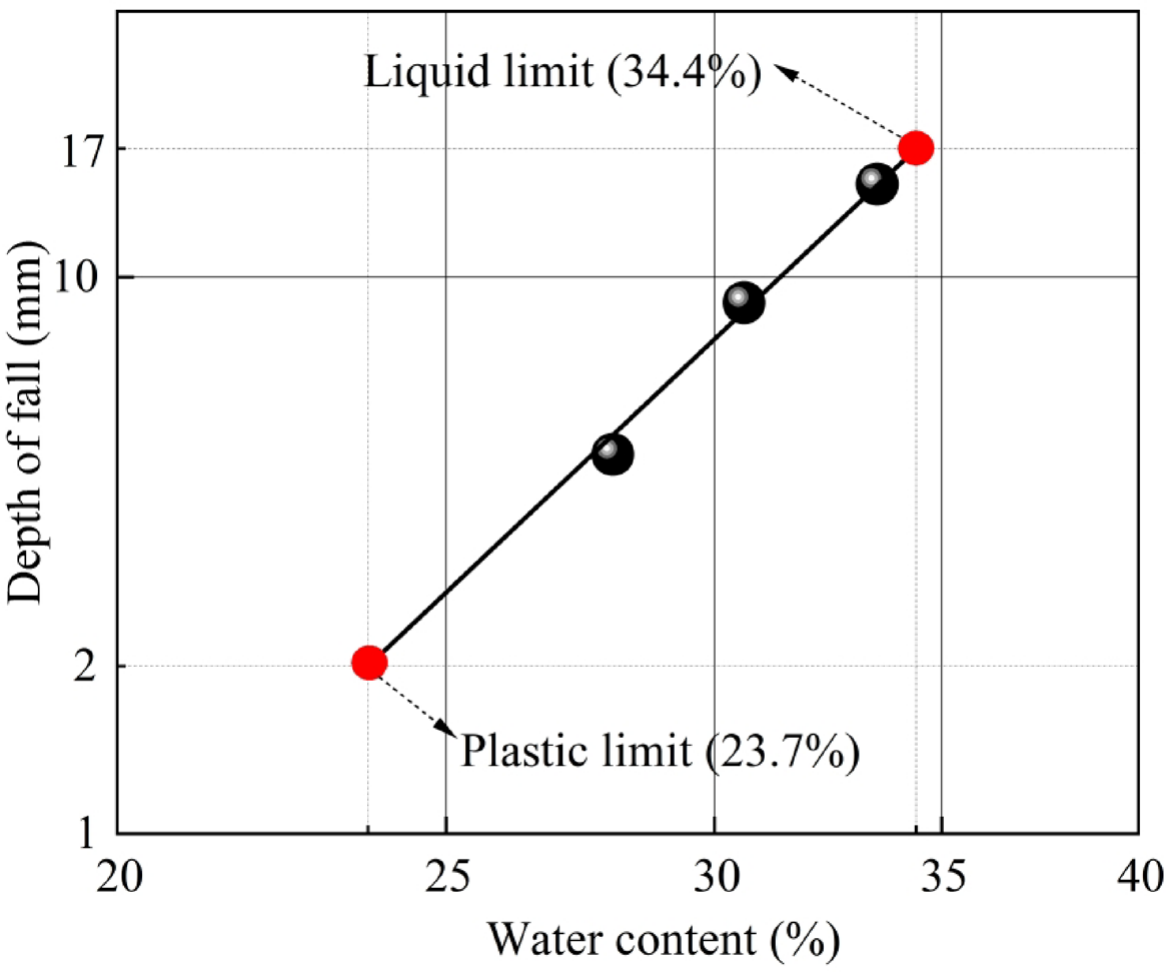
Liquid limit and plastic limit.

The relationship curve between cone penetration depth and water content was derived using a combined liquid and plastic limit apparatus (Fig. 2). The plastic limit of the soil sample was determined to be 23.7%, the liquid limit was 34.4%, and the plasticity index was 10.7%. According to Chinese standard GB50007-2011 “Code for design of building foundation”, soils with plasticity indices between 10 and 17% should be classified as silty clay. Consequently, the soil sampled from the Xing’an Baikal permafrost area in this study is classified as silty clay.

**Fig. 3:**
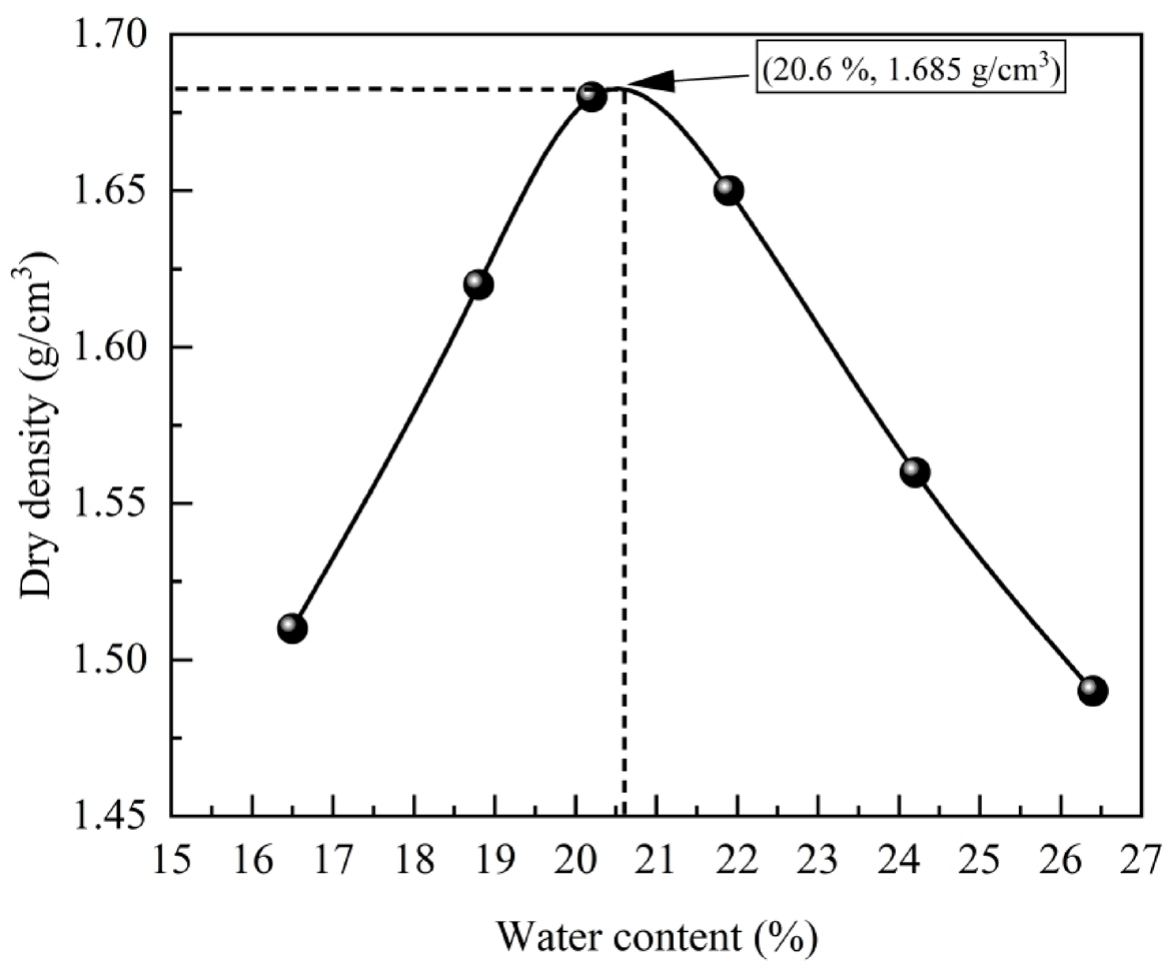
Compaction curve.

The compaction curve for the soil sample was derived from a lightweight compaction test (Fig. 3). The results indicate that the optimal water content for the silty clay from the Daxing’anling is 20.6%, accompanied by a maximum dry density of 1.685 g/cm^3^.

### Methods

**Fig. 4:**
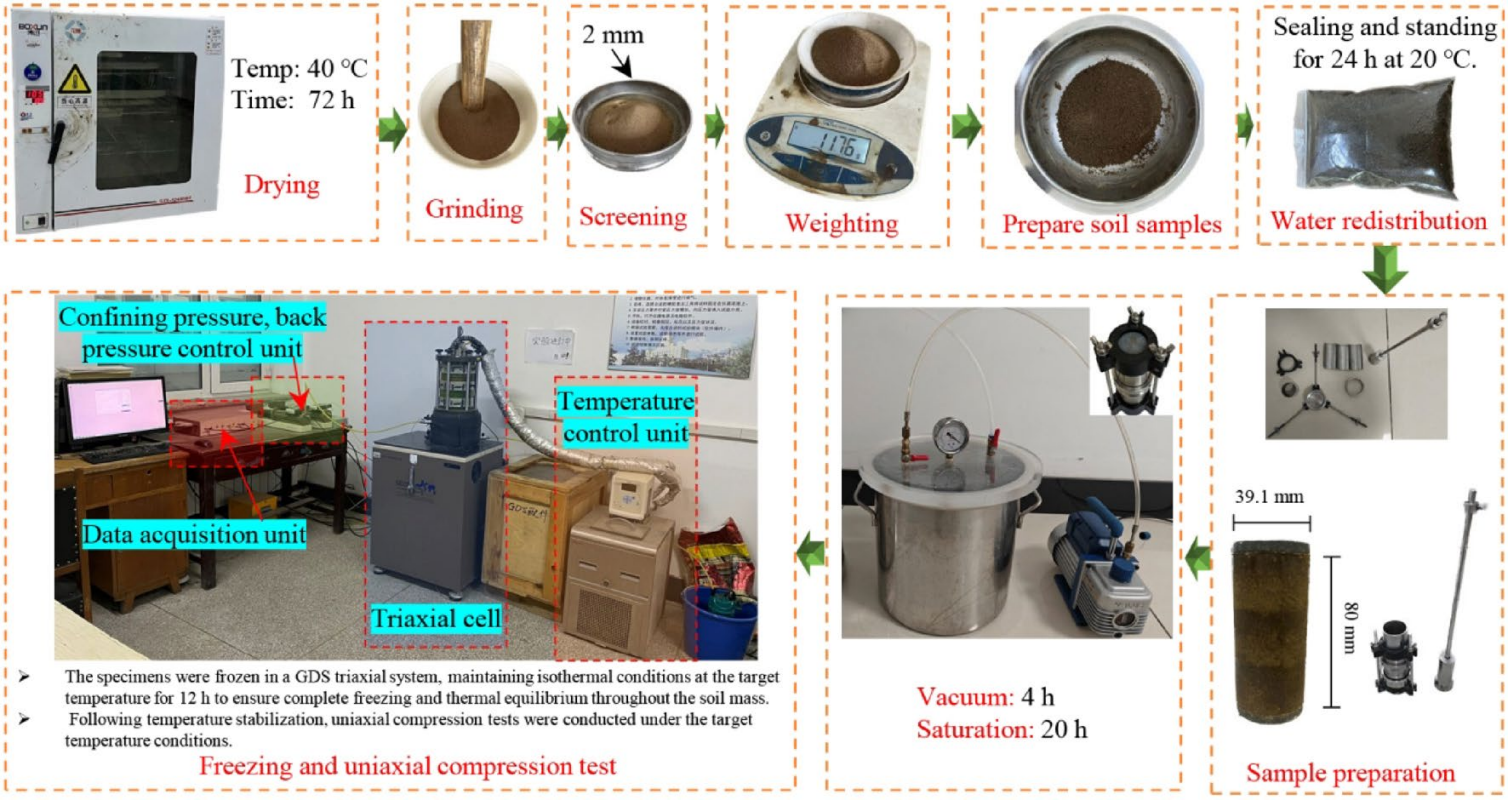
Experimental roadmap.

As illustrated in Fig. 4, the soil samples underwent specialized pretreatment to preserve their geotechnical properties. To avoid damaging interparticle cementation or decomposing organic matter that typically occurs at elevated temperatures (e.g., 105 °C), samples were first dried at a controlled low temperature of 40 °C for 72 h, effectively removing free water while minimizing impacts on clay minerals and preserving the native soil structure. The dried soil was then carefully ground to disperse aggregates without disrupting chemical bonds between particles, followed by sieving through a 2 mm mesh to remove coarse grains and ensure sample homogeneity while preventing potential stress concentration effects during testing. Based on Proctor compaction test results showing a maximum dry density of 1.685 g/cm^3^, test specimens were prepared at 95% compaction to simulate actual engineering conditions, formed to triaxial dimensions of *φ*39.1 × 80 mm using distilled water to eliminate ionic interference. After thorough mixing, the samples were sealed and cured at 20 °C for 24 h to achieve uniform moisture distribution^25^, then compacted using layered compaction techniques. Finally, vacuum saturation was performed through 4 h evacuation followed by 20 h water immersion to ensure saturation levels exceeding 95% for subsequent testing.

Upon completion of vacuum saturation, the specimens were placed in a GDS triaxial apparatus for freezing tests. The freezing process employed a programmable temperature control system, with the target temperature being continuously monitored by thermocouples installed inside the pressure chamber of the GDS triaxial system. Once the chamber temperature reached the target value, it was maintained constant for 12 h to ensure complete freezing of the soil specimen and stabilization of its internal temperature. Following temperature stabilization, uniaxial compression tests were conducted at subzero temperatures while maintaining the target temperature condition. Throughout the testing process, constant temperature was maintained using the triaxial system’s built-in refrigeration unit combined with an external circulating fluid system.

Despite these measures, some temperature management uncertainties may persist. For instance, localized heat exchange at the interface between the specimen and loading platen could result in slightly lower boundary temperatures compared to the core. Additionally, transient temperature fluctuations may occur during freezing due to latent heat release from the water–ice phase transition.

Once the soil temperature stabilized, uniaxial compression tests were conducted under negative temperature conditions using the GDS triaxial apparatus. The tests were conducted under unconsolidated undrained (UU) conditions at a shear rate of 0.8 mm/min, and the tests were terminated when the axial strain reached 20%. Based on the temperature monitoring results of permafrost in the research area^9–12^, mechanical tests are scheduled for the samples at temperatures of − 0.5 °C, − 1.0 °C, − 2.0 °C, − 3.0 °C, − 5.0 °C, and − 7.5 °C.

### Data processing

#### Compression strength

**Fig. 5:**
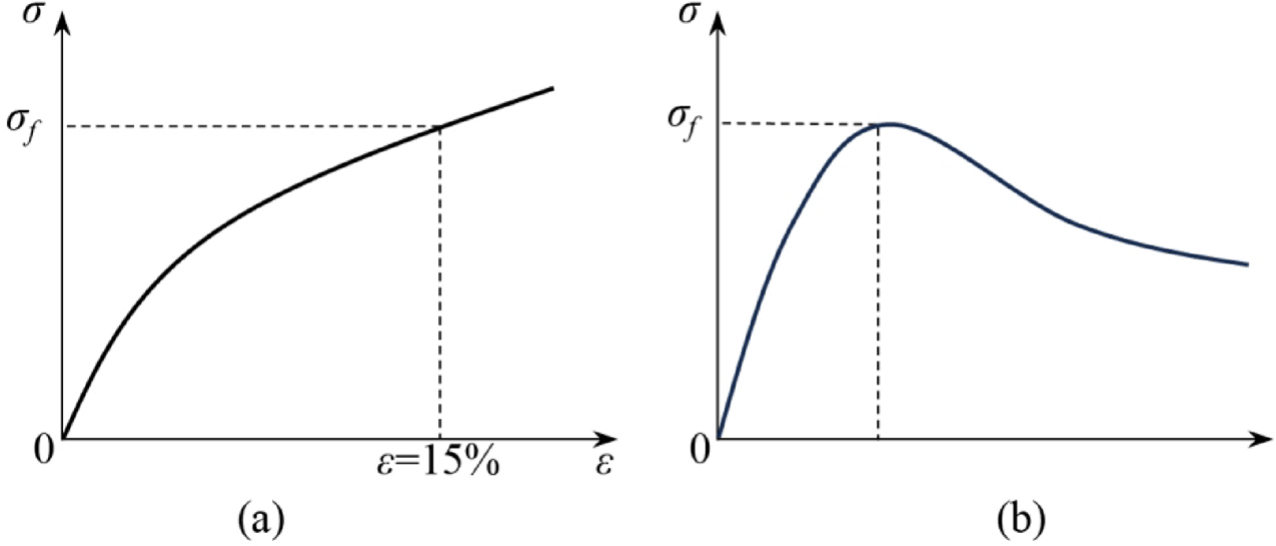
Compression strength.

For different types of stress–strain curves, the failure stress is identified in distinct manners: the strain-hardening behavior stress–strain curve (Fig. 5a) exhibits a continuous increase in stress with increasing strain, without a distinct stress peak^26^. Consequently, the stress at 15% strain is regarded as the compression strength^27^. In contrast, the strain-softening behavior stress–strain curve (Fig. 5b) indicates that stress initially increases and subsequently decreases with increasing strain, exhibiting a clear stress peak^28^. Consequently, this peak value is taken directly as the compression strength^27^.

#### Secant modulus

In materials mechanics, the elastic modulus is generally considered a crucial parameter for analyzing material deformation and stability^29,30^. In geotechnical studies, soil deformation encompasses both recoverable elastic deformation and irreversible non-elastic deformation^31^. When investigating the deformation modulus in geotechnics, emphasis is typically placed on the secant modulus and tangent modulus^32^.

**Fig. 6:**
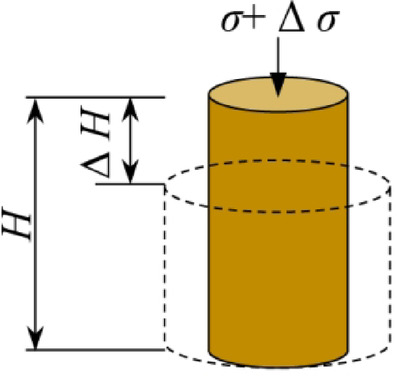
The schematic of soil deformation.

In geotechnical studies, the secant modulus is frequently utilized to characterize the axial deformation of soil during shear. As illustrated in Fig. 6, when a force is applied vertically to the soil sample, strain occurs in the vertical direction. The ratio of the increment in stress to the increment in strain at this point is defined as the secant modulus (Eq. 1)^33^.

1 $$\it {\text{E}}_{\sec } = {{\Delta \sigma } \mathord{\left/ {\vphantom {{\Delta \sigma } {\Delta \varepsilon }}} \right. \kern-0pt} {\Delta \varepsilon }}$$

where *E*_*sec*_ represents the secant modulus, Δ*σ* is the increment in stress, and Δ*ε* is the increment in strain.

#### Initial tangent modulus

**Fig. 7:**
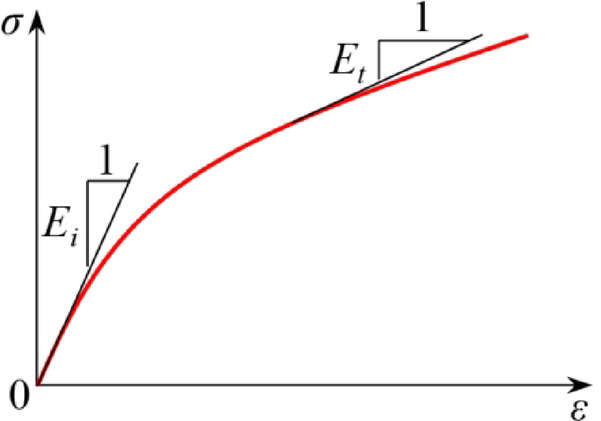
Tangent modulus and initial tangent modulus.

As illustrated in Fig. 7, when the increment in stress approaches infinitesimal values, the ratio of the increment in stress to the increment in strain is defined as the tangent modulus (Eq. 2).

2 $$\it {\text{E}}_{t} = {{d\sigma } \mathord{\left/ {\vphantom {{d\sigma } {d\varepsilon }}} \right. \kern-0pt} {d\varepsilon }}$$

where *E*_*t*_ represents the tangent modulus, d*σ* is the increment in stress, and d*ε* is the increment in strain.

During the shear process, the value of the tangent modulus diminishes with increasing stress. At the initial stage of shear, the tangent modulus reaches its maximum value, defined as the initial tangent modulus. In this study, the initial tangent modulus is determined as the ratio of the increment in stress to the increment in axial strain when the axial strain of the sample reaches 2.0% on the stress–strain curve^34^.

## Results

### Changes in the stress–strain curves

**Fig. 8:**
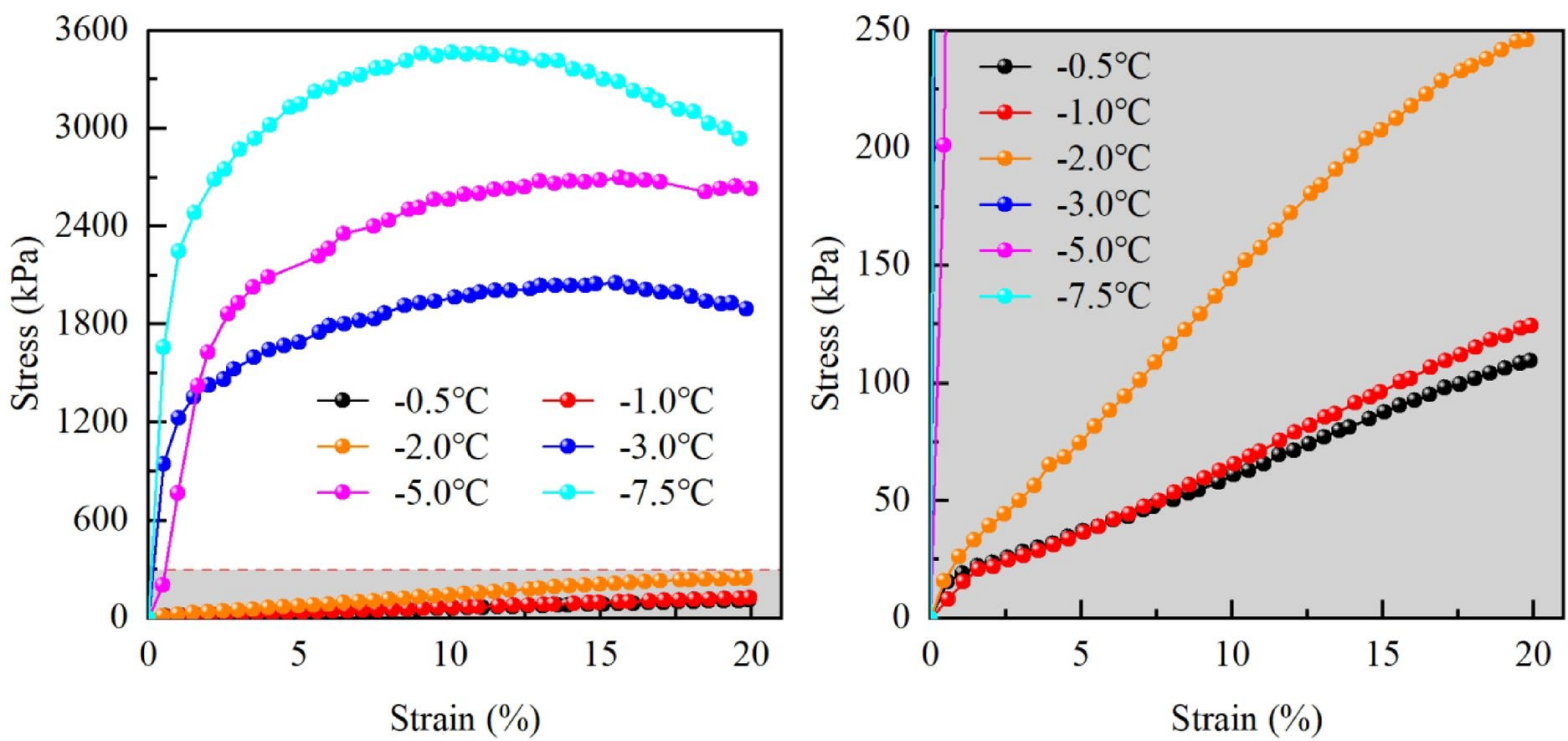
Stress–strain curves.

Figure 8 illustrates the stress–strain curves of silty clay in the Xing’an Baikal permafrost region at various freezing temperatures. The stress–strain curve of low-temperature frozen soil demonstrates strain-softening behavior with a distinct yield point. As the soil temperature increases, the classification of frozen soil transitions from low-temperature to high-temperature. In uniaxial compression tests, the stress–strain curve transitions from strain-softening to strain-hardening, and the yield point becomes less pronounced. During this transition from low-temperature to high-temperature frozen soil, the unfrozen-water content in the frozen soil continues to increase until surpassing the phase change zone, after which unfrozen water remains in the soil. The increasing unfrozen-water content correlates with a decrease in ice content, diminishing the cementing effect between soil particles, which results in a reduction in soil strength. Consequently, the failure mode of the soil transitions from brittle failure to plastic failure during uniaxial compression tests.

### Changes in the secant modulus

**Fig. 9:**
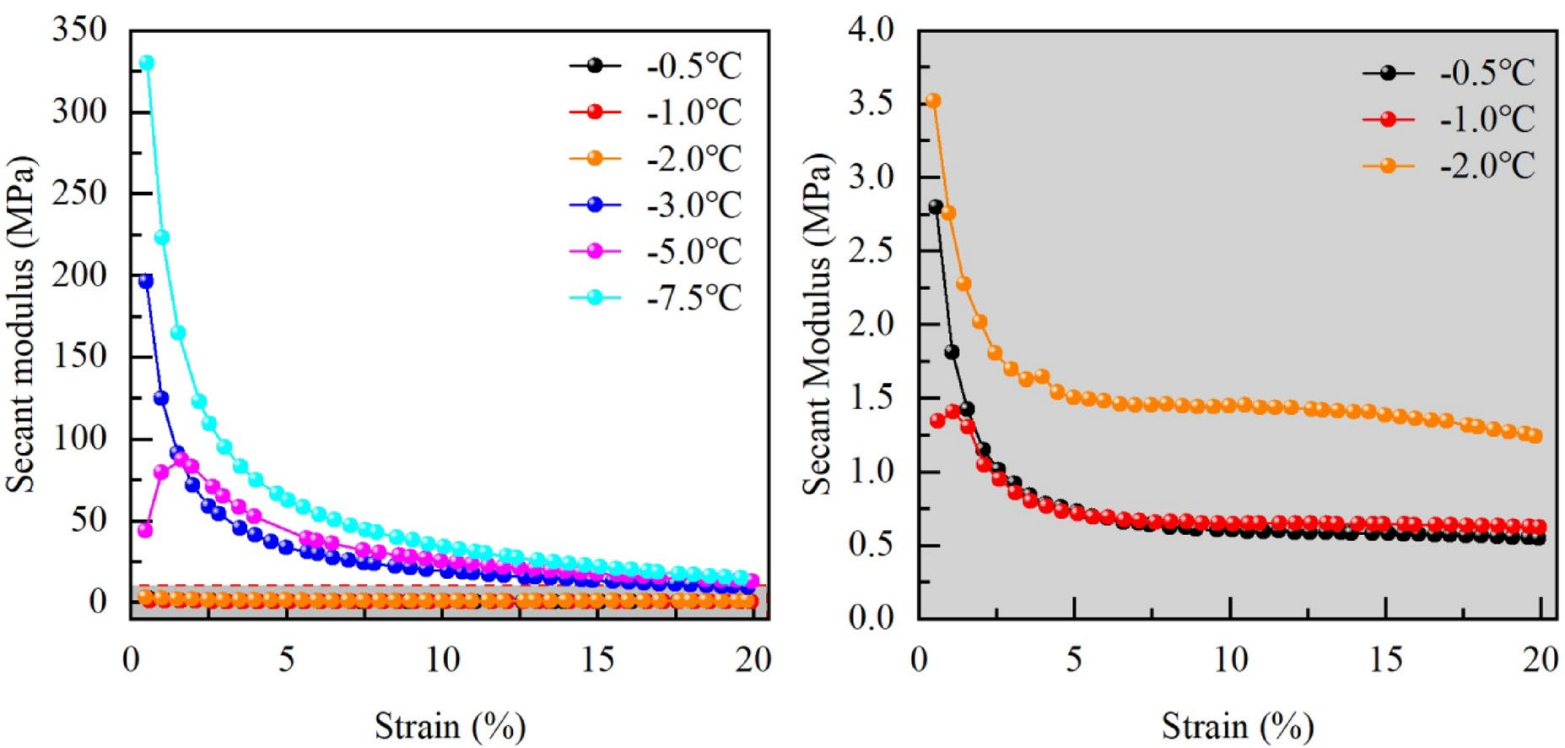
Changes of secant modulus.

The secant modulus of frozen silty clay in the Xing’an Baikal permafrost region at various temperatures was calculated based on Eq. (1) and the stress–strain curves obtained from uniaxial tests, as illustrated in Fig. 9.

Figure 9 illustrates that the secant modulus-strain curves of frozen silty clay at various temperatures generally exhibit a pattern of decreasing secant modulus with increasing axial strain. However, at − 5.0 °C and − 1.0 °C, the curves initially demonstrate an increase in secant modulus with axial strain. Ultimately, they align with the trend of decreasing secant modulus with increasing axial strain, stabilizing at lower values. As temperature increases, the strain required for the secant modulus to stabilize decreases, while the corresponding secant modulus at stabilization for low-temperature frozen soil remains higher.

### Changes in the initial tangent modulus

**Fig. 10:**
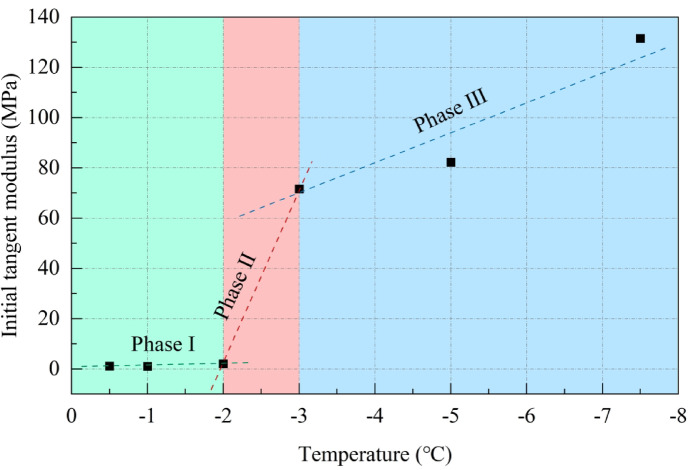
Changes of initial tangent modulus.

Figure 10 illustrates the trend of the initial tangent modulus of frozen silty clay in the Xing’an Baikal permafrost region as it varies with temperature at different freezing temperatures.

Figure 10 illustrates the trend of the initial tangent modulus of silty clay at various freezing temperatures as it varies with temperature. The initial tangent modulus of frozen silty clay demonstrates a negative correlation with temperature. Based on the trend of the initial tangent modulus, three distinct stages can be identified: Stage I, where the initial tangent modulus slowly increases as the temperature decreases from − 0.5 to − 2.0 °C, rising from 1.18 to 2.00 MPa at a rate of 0.61 MPa/°C; Stage II, a rapid growth phase, where the temperature decreases from − 2.0 to − 3.0 °C, and the initial tangent modulus increases from 2.00 to 71.58 MPa at a rate of 69.58 MPa/°C; and Stage III, a slow growth phase, where the temperature decreases from − 3.0 to − 7.5 °C, and the initial tangent modulus rises from 71.58 to 131.52 MPa at a rate of 13.32 MPa/°C. The variation in the initial tangent modulus suggests that lower temperatures are more favorable for the structure and stability of the soil; under load, high-temperature frozen soil is more susceptible to instability compared to low-temperature frozen soil.

### Changes in the compression strength

**Fig. 11:**
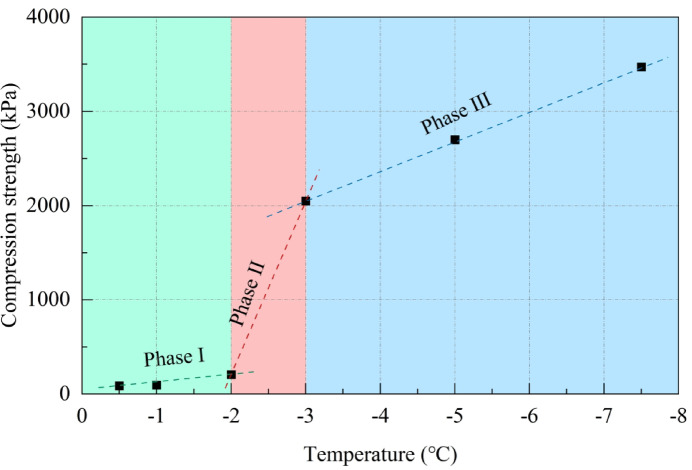
Changes of compression strength.

In this study, the stress–strain curve of high-temperature frozen soil demonstrates strain-hardening behavior, while that of low-temperature frozen soil exhibits strain-softening behavior. For the strain-hardening high-temperature frozen soil, the stress corresponding to an axial strain of 15% is designated as the compression strength. For the strain-softening low-temperature frozen soil, the peak stress is directly considered as the compression strength. The variation in compression strength of frozen silty clay at different temperatures is illustrated in Fig. 11.

Similar to the trend observed in the initial tangent modulus, the compression strength of frozen silty clay demonstrates a negative correlation with temperature. As the temperature decreases, the compression strength undergoes three distinct phases of change: Phase I demonstrates a gradual increase in compression strength, with the temperature decreasing from − 0.5 to − 2.0 °C, resulting in an increase from 87.78 to 207.77 kPa, at a rate of 79.99 kPa/°C; Phase II is characterized by a rapid increase, with the temperature decreasing from − 2.0 to − 3.0 °C, resulting in an increase from 207.77 to 2049.77 kPa, at a rate of 1842.00 kPa/°C; Phase III demonstrates a slow increase, with the temperature decreasing from − 3.0 to − 7.5 °C, resulting in an increase from 2049.77 to 3472.66 kPa, at a rate of 316.20 kPa/°C.

### Statistical analysis

**Fig. 12:**
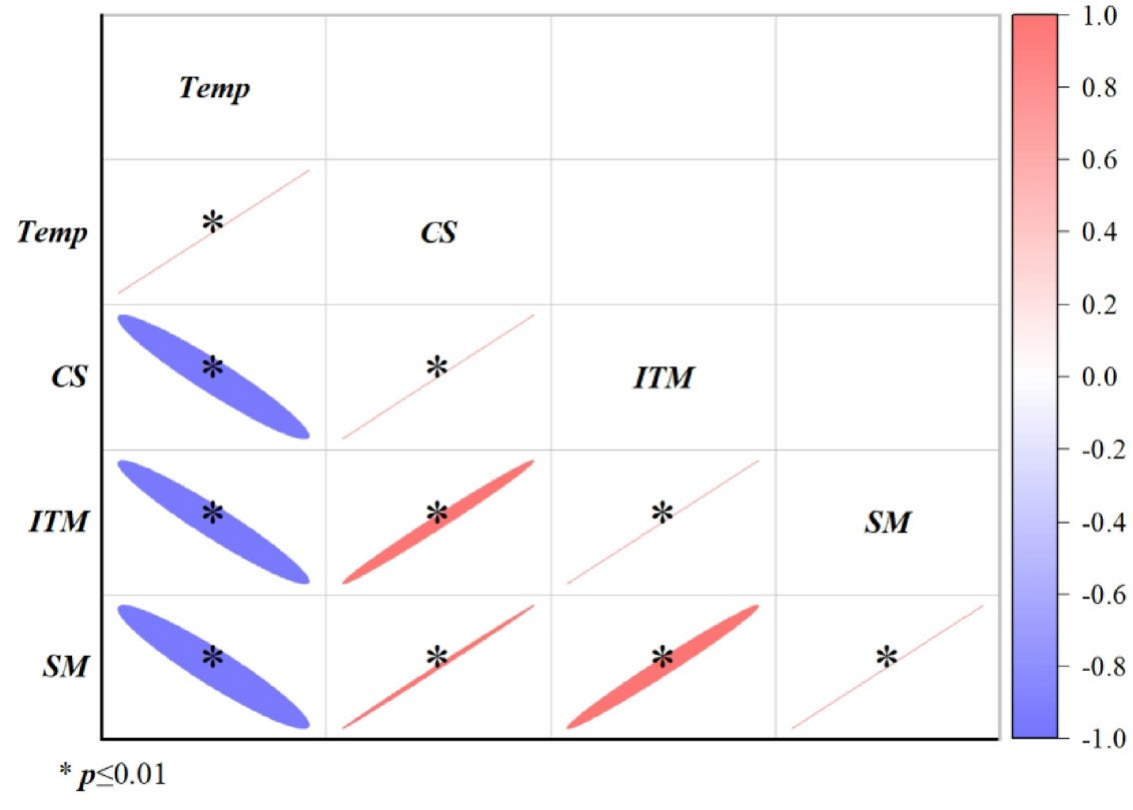
Pearson’s correlation and significance between temperature and the strength of frozen silty clay in the Xing’an Baikal permafrost region (*Temp*: temperature; *CS*: compression strength; *ITM*: initial tangent modulus; *SM*: secant modulus).

Figure 12 presents the Pearson correlation analysis between temperature and the mechanical properties of frozen silty clay from the Daxing’anling, revealing statistically significant negative correlations (*p* ≤ 0.01) with compressive strength, initial tangent modulus, and secant deformation modulus. These results demonstrate that as temperature decreases, all three mechanical parameters exhibit significant enhancement.

## Discussions

### Relationships among temperature, unfrozen water content, ice content, and frozen soil strength

The results from uniaxial compression tests clearly indicate that temperature significantly influences the strength of frozen silty clay. Furthermore, the variation in soil strength exhibits significant differences across various temperature ranges. Findings from relevant literature suggest that the strength of frozen soil is closely related to the ice morphology within the soil and the content of unfrozen water^35,36^.

**Fig. 13:**
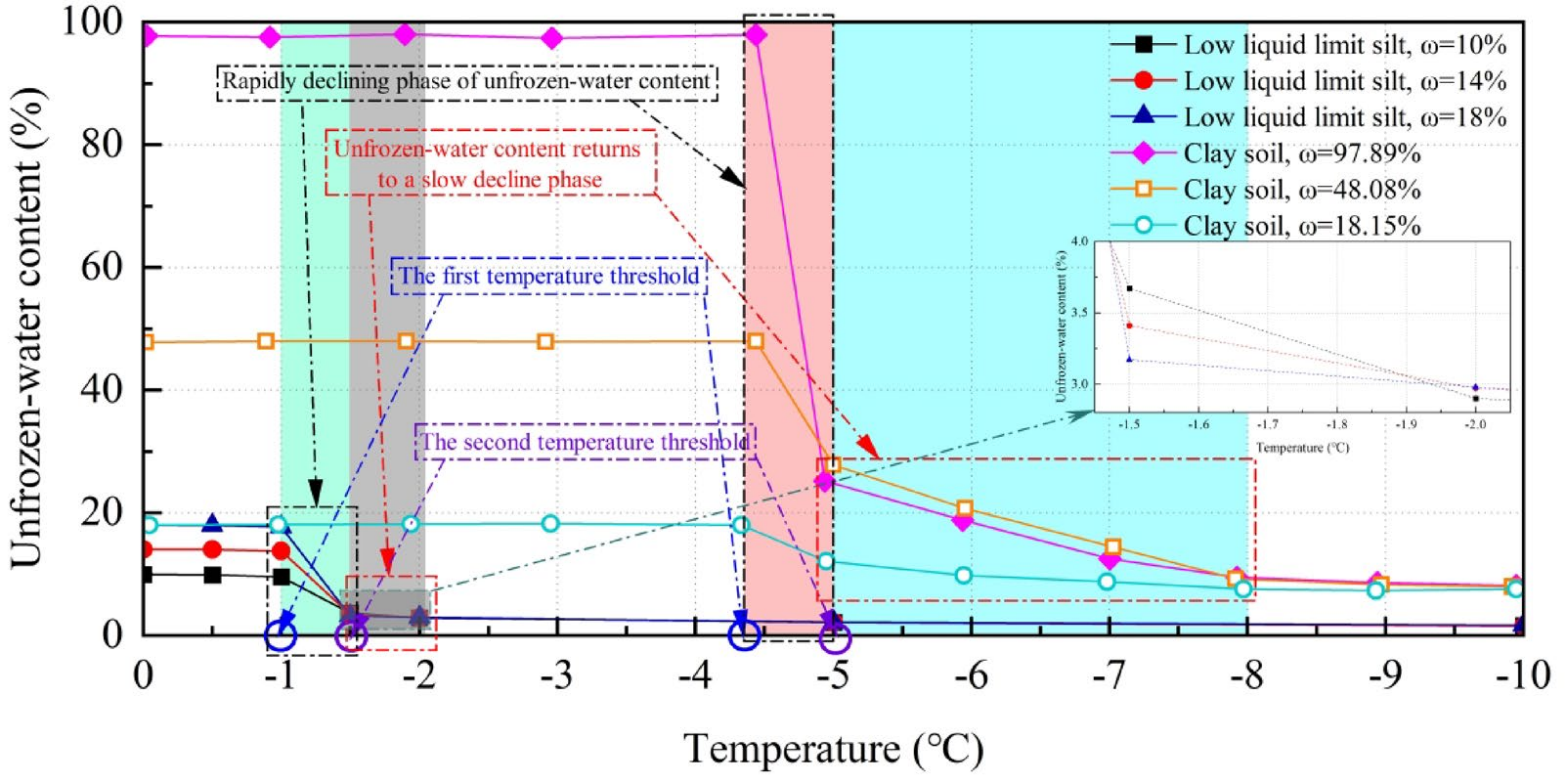
The relationship between unfrozen water content and soil freezing temperature. *Notes*: The data for low liquid limit silt (Shuozhou-Huanghua Railway) is obtained from the study by Zhang et al.^37^, while the data for clay soil (Harbin) is derived from the research by Yang et al.^38^. Additionally, ω represents the initial moisture content of the soil.

Due to the constraints of the experimental conditions, this study was unable to investigate the relationship between soil temperature and unfrozen-water content from an experimental perspective. Instead, this study references existing research to summarize the relationship between unfrozen-water content and soil temperature (Fig. 13), ultimately elucidating the connection between temperature and the strength of frozen silty clay.

**Fig. 14:**
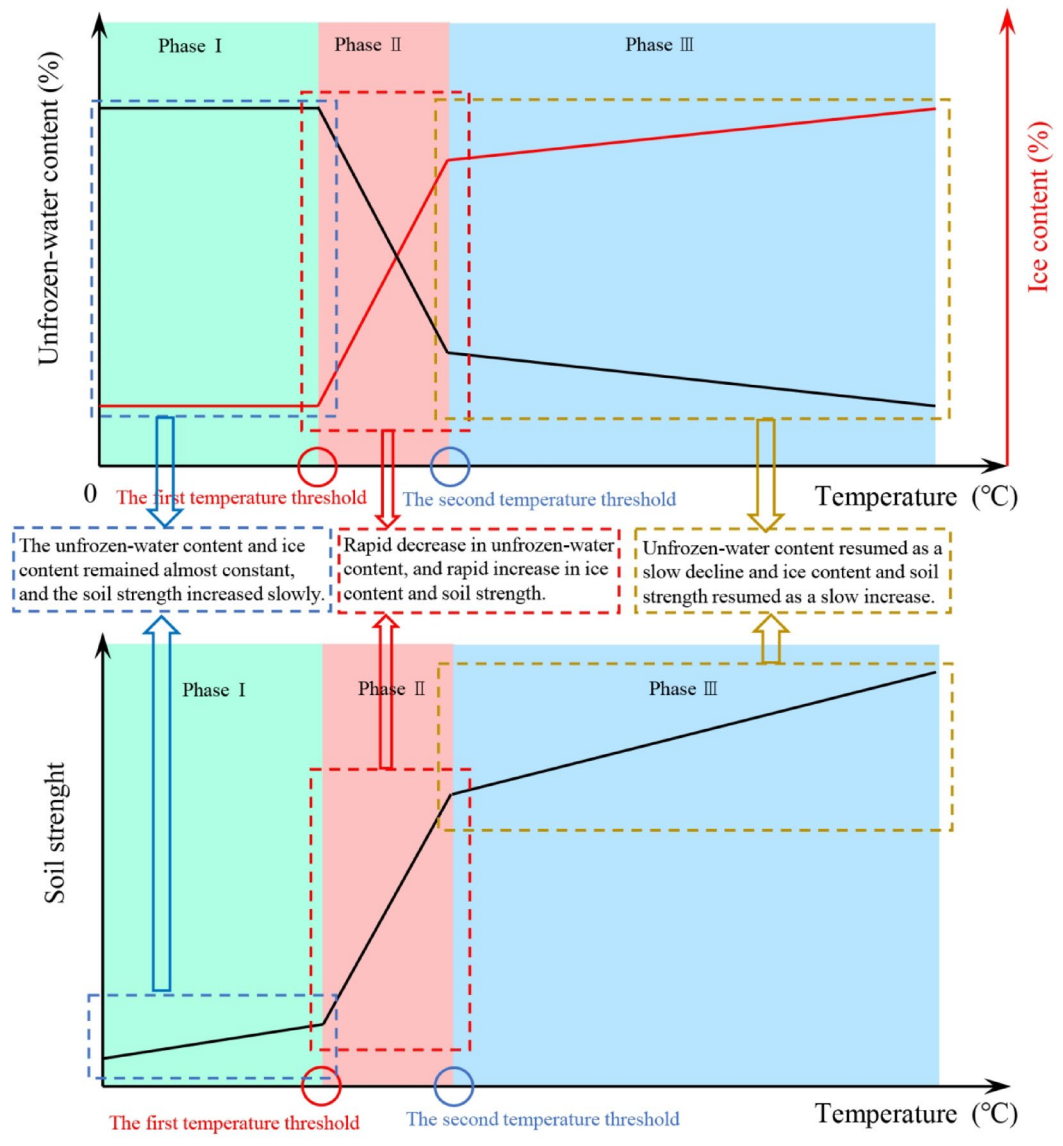
The relationship between temperature, unfrozen-water content, ice content and soil strength.

Figure 14 illustrates that as the soil temperature decreases, both the low liquid limit silt from the Shuozhou-Huanghua Railway and the clay from Harbin demonstrate a distinct “three-stage” trend in unfrozen-water content during the freezing process:

*Stage I*: The unfrozen-water content remains relatively constant as the temperature decreases.

*Stage II*: As the temperature continues to decline, once it reaches the first threshold, water in the soil rapidly freezes, resulting in a swift decrease in unfrozen-water content. This process occurs almost instantaneously due to the principles of supercooled water forming ice crystals, even with minimal temperature changes.

*Stage III*: After the temperature further declines to the second threshold, water in the soil slowly freezes into ice, resulting in minimal changes in unfrozen-water content. The rate of decrease in unfrozen-water content diminishes, ultimately stabilizing.

The unfrozen-water content is inversely related to the ice content in the soil, and the variations in ice content can also be categorized into three stages:

*Stage I*: As the temperature decreases, the ice content is 0 and remains unchanged.

*Stage II*: With the continuous decline in temperature, once it reaches the first threshold, the ice content increases rapidly.

*Stage III*: After the temperature further decreases to the second threshold, the ice content rises slowly, ultimately resulting in the stabilization of unfrozen-water content.

Based on the aforementioned theoretical research regarding the relationships among unfrozen-water content, ice content, and soil strength, the correlation between the strength of frozen silty clay in the Xing’an Baikal permafrost region and temperature can be analyzed (Fig. 14).

As the temperature decreases, during the initial stage (Stage I), the unfrozen-water content remains nearly constant, resulting in an ice content of 0. However, as the temperature decreases, the viscosity of water increases^39^, generating resistance to the movement of soil particles. This is reflected in the gradual increase of various strength parameters of the soil during mechanical tests. When the temperature reaches the first threshold, Stage II begins, characterized by a rapid phase change of water. During this phase change, most of the liquid water converts to solid ice, while a significant amount of unfrozen water persists. The soil comprises a mixture of soil skeleton, solid ice, and unfrozen water. In this process, ice begins to function as a binding agent between soil particles, resulting in a substantial increase in soil strength. This phase is associated with a rapid increase in the strength parameters of frozen silty clay in the Daxing’anling. Once the temperature reaches the second threshold, Stage III begins, during which the unfrozen-water content gradually decreases with further temperature reduction, while the ice content increases, enhancing the binding effect between soil particles. However, the rates of decrease in unfrozen-water content and increase in ice content both slow, resulting in a diminishing rate of strength increase for the soil.

### Soil-ice interface characteristics governing frozen soil strength

**Fig. 15:**
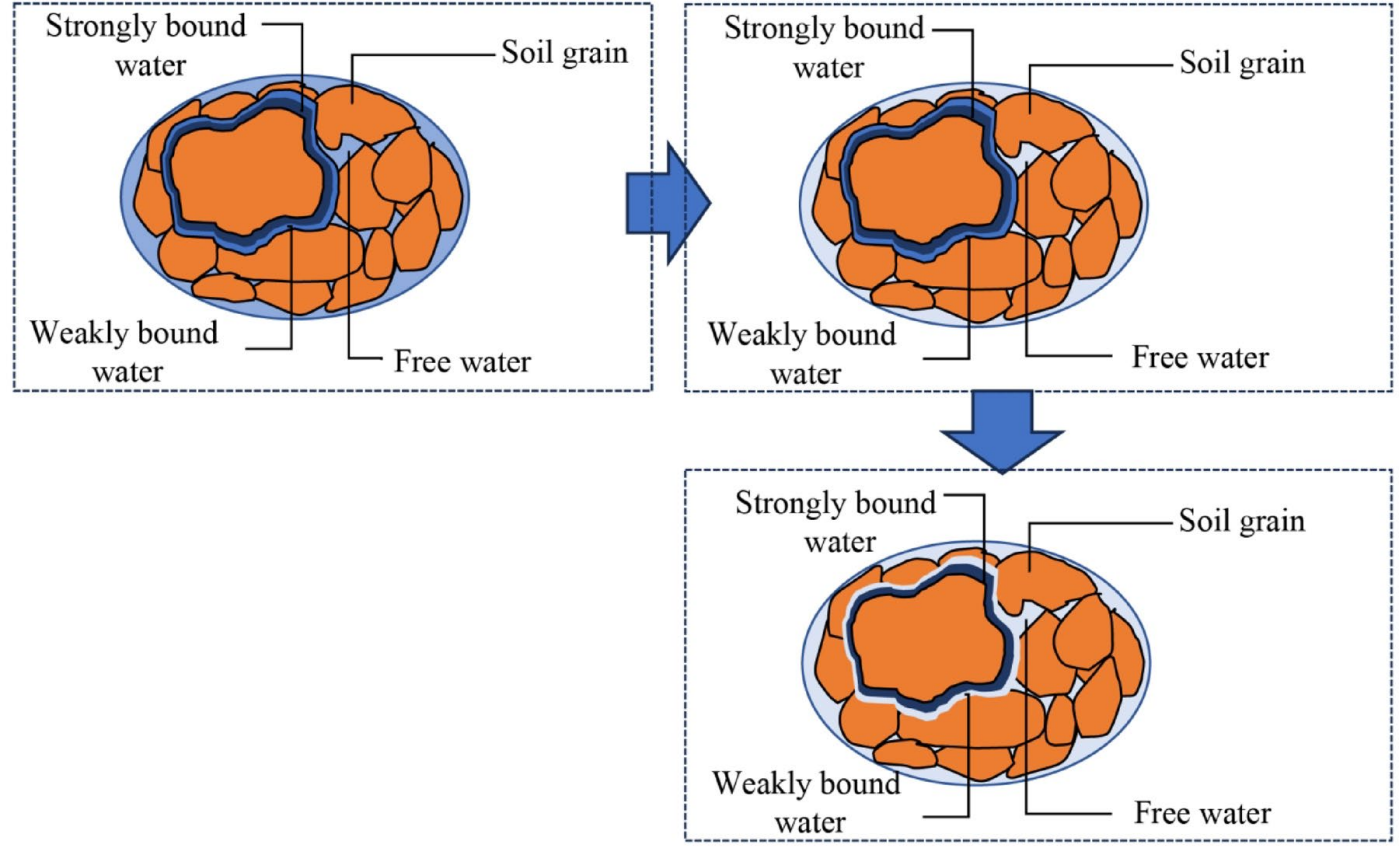
Freezing process of water in soil.

During soil cooling, different water types exhibit distinct phase transitions. As shown in Fig. 15, in unfrozen soil, free water, weakly bound water, and strongly bound water all exist in liquid form between soil particles. As temperature decreases and freezing initiates, pore free water first solidifies into ice, while weakly and strongly bound water remain liquid around particles. With further cooling, weakly bound water undergoes liquid–solid phase transition^40^, whereas strongly bound water persists as liquid films coating particle surfaces. Since the freezing behavior of strongly bound water remains controversial, this study excludes it from the freezing process analysis.

**Fig. 16:**
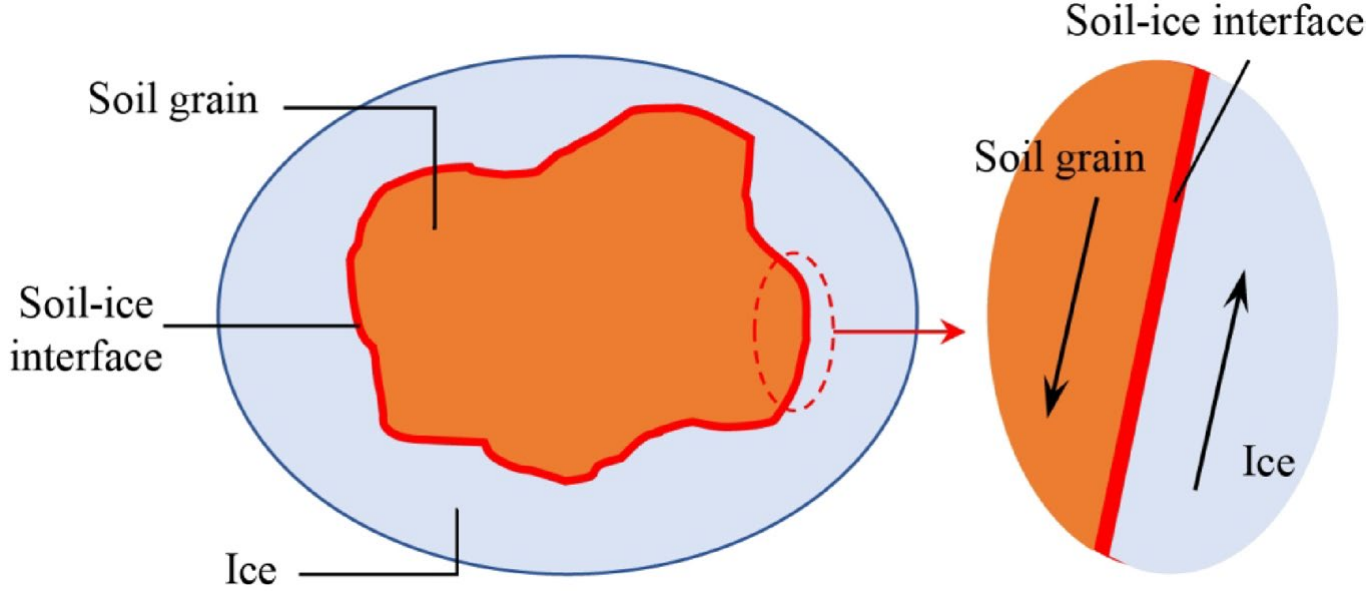
Schematic diagram of soil-ice interface.

Analysis of the water freezing process in frozen soils reveals the existence of a soil-ice interface between soil particles and ice (Fig. 16). As two distinct materials, the soil-ice interface forms a structural weak plane in frozen soil. Under loading, shear failure preferentially occurs along this interface.

At higher temperatures, the soil-ice interface consists of both strongly and weakly bound water. The weakly bound water, experiencing weaker electric field forces, facilitates failure under relatively small loads. As temperature decreases and weakly bound water freezes, only strongly bound water remains at the interface. The stronger electric field forces acting on strongly bound water increase resistance to deformation, requiring substantially higher loads to induce failure.

### Analysis of temperature dependence of initial tangent modulus

The three-stage evolution of the initial tangent modulus (Fig. 10) is fundamentally governed by the coupled effects of unfrozen water phase transitions, soil-ice interface reorganization, and ice framework reinforcement: (1) In the − 0.5 to − 2.0 °C range, abundant unfrozen water provides interparticle lubrication, with modulus enhancement primarily driven by increasing water film viscosity; (2) Between − 2.0 °C and − 3.0 °C, massive unfrozen water freezing transforms interfaces to bound water-dominated states while forming continuous ice skeletons, resulting in rapid modulus escalation; (3) Below − 3.0 °C, further unfrozen water reduction establishes strongly bound water interfaces that sustain—but progressively slow—modulus increases due to diminishing phase change contributions.

### Implications of the findings for engineering applications in cold regions

These findings provide critical guidance for the design, construction, and maintenance of cold-region engineering projects (e.g., roads, bridges, pipelines, and buildings in the Daxing’anling and similar high-latitude permafrost regions).

Frozen soil strength exhibits a three-phase variation (slow → rapid → slow increase) with decreasing temperature, with the most rapid strength gain occurring between − 2.0 and − 3.0 °C. Consequently, during cold seasons, the higher bearing capacity of frozen soil allows temporary utilization of its strength for construction. However, as temperatures approach the phase transition point (− 0.5 to − 2.0 °C), strength declines significantly, necessitating precautions against foundation thaw settlement, slope instability, and other hazards. Enhanced monitoring or insulation measures (e.g., thermal insulation layers) are required in such cases.

Reduced unfrozen water content at low temperatures shifts soil failure from plastic to brittle, requiring foundation designs to account for sudden frozen soil fracture risks.

The initial tangent modulus exhibits markedly different temperature-dependent behavior across freezing ranges, with an exceptionally rapid increase of 69.58 MPa/°C between − 2.0 and − 3.0 °C, compared to much slower growth rates in other temperature intervals. This nonlinear characteristic necessitates temperature-dependent segmentation in engineering calculations rather than simplified linear approximations. Furthermore, seasonal load adjustments must be implemented—for instance, permitting higher traffic loads during winter months while enforcing strict load restrictions in spring when the permafrost undergoes phase transitions.

### Damage model

#### Damage variables

In contemporary theoretical research on material damage mechanics, damage variables are typically defined in terms of the area of material defects, changes in ultrasonic wave velocity, and variations in elastic modulus^41,42^.

1. Definition of damage variables based on the area of material defects:

3 $$ {\it\text{D}} = 1 - {{A^{\prime } } \mathord{\left/ {\vphantom {{A^{\prime } } A}} \right. \kern-0pt} A}$$

where *A* represents initial area of the material; *A*′ is effective area of the material after damage.

Assuming the nominal stress corresponding to the initial area *A* of the material is *σ*, and the effective stress corresponding to the effective area *A*′ after damage is *σ*′, Eq. (3) can be expressed as:

4 $${\it\text{D}} = 1 - {{A^{\prime } } \mathord{\left/ {\vphantom {{A^{\prime } } A}} \right. \kern-0pt} A} = 1 - {{\sigma^{\prime } } \mathord{\left/ {\vphantom {{\sigma^{\prime } } \sigma }} \right. \kern-0pt} \sigma }$$

(2) Definition of the damage variable based on changes in ultrasonic wave velocity:

When ultrasonic waves pass through a material, they interact with it, and the presence of cracks affects the wave velocity. Therefore, the damage variable can be defined based on changes in ultrasonic wave velocity:

5 $${\it \text{D}} = 1 - {\it {{\text{ v}}^{\prime } } \mathord{\left/ {\vphantom {{{\text{v}}^{\prime } } {\text{v}} }} \right. \kern-0pt} {\text{v}} }$$

where *v* represents wave velocity of the damaged material; *v’* is wave velocity of the material after damage.

**Fig. 17:**
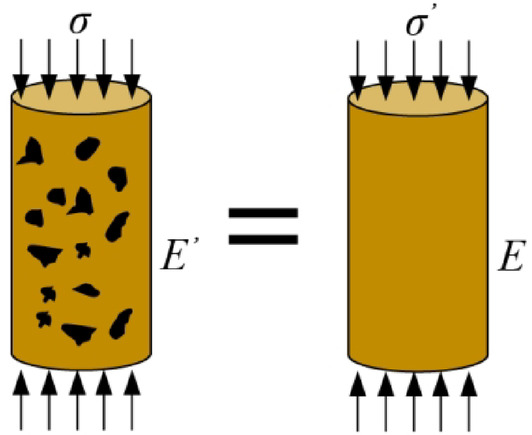
Schematic of Lemaitre assumption.

(3) Definition of the damage variable based on changes in material elastic modulus:

In practical applications, obtaining the effective area directly through macro-scale testing is challenging, while wave velocity must be measured using ultrasonic testing devices. To address this challenge, a damage variable based on changes in the elastic modulus has been proposed. The widely accepted Lemaitre assumption posits that the strain induced by stress on damaged material is equivalent to the strain produced by the same stress on undamaged material (Fig. 17)^43^.

The mechanical damage of frozen soil under loading primarily manifests as elastic modulus degradation, consistent with the core premise of Lemaitre’s strain equivalence hypothesis that damage can be characterized through modulus reduction. This approach allows direct determination of the damage variable by comparing undamaged and damaged elastic moduli, circumventing the need for complex microstructural analysis. Alternative damage theories show limitations when applied to frozen soils: The Kachanov-Rabotnov effective area hypothesis, while useful for describing damage through effective stress concepts^44^, requires precise micro-defect geometry characterization—a challenge for frozen soils where damage develops via ice crystal fracture and pore restructuring rather than discrete crack propagation. Similarly, Mazars’ anisotropic damage model^45^, though effective for many engineered materials, introduces excessive parameters (e.g., damage tensors) that typically exceed what can be reliably determined from conventional frozen soil test data. These considerations make Lemaitre’s hypothesis particularly suitable, as its macroscopic statistical characterization aligns well with frozen soil’s heterogeneous damage mechanisms while maintaining experimental practicality through modulus-based quantification and avoiding the parameter over-specification common to more complex models.

According to the Lemaitre assumption, the constitutive relationship of damaged material can be derived from the nominal stress in undamaged material, specifically:

6 $$\varepsilon = {\sigma \mathord{\left/ {\vphantom {\sigma {{\text{E}}^{\prime } }}} \right. \kern-0pt} {{\it \text{E}}^{\prime } }} = {{\sigma^{\prime } } \mathord{\left/ {\vphantom {{\sigma^{\prime } } {\text{E}} }} \right. \kern-0pt} {\it \text{E}} } = {\sigma \mathord{\left/ {\vphantom {\sigma {\left[ {{\text{E}} \left( {1 - {\text{D}} } \right)} \right]}}} \right. \kern-0pt} {\left[ {{\it \text{E}} \left( {1 - {\it \text{D}} } \right)} \right]}}$$

where *E* represents the elastic modulus of undamaged material, also referred to as the initial elastic modulus; *E*′ is the elastic modulus of damaged material, known as the effective elastic modulus.

By combining Eqs. (4) and (6), all forms of the damage variable can be obtained.

7 $$ {\it\text{D}} = 1 - {{\sigma^{\prime } } \mathord{\left/ {\vphantom {{\sigma^{\prime } } \sigma }} \right. \kern-0pt} \sigma } = 1 - {{A^{\prime } } \mathord{\left/ {\vphantom {{A^{\prime } } A}} \right. \kern-0pt} A} = 1 - {{E^{\prime } } \mathord{\left/ {\vphantom {{E^{\prime } } E}} \right. \kern-0pt} E}$$

Additionally, Eq. (6) can be rearranged as follows:

8 $$ \sigma = {\it\text{E}} \varepsilon \left( {1 - {\it\text{D}} } \right)$$

#### Determination of damage variables

For frozen silty clay subjected to increased temperature and loading, the damage variable comprises two components. The first component, the temperature-induced damage variable *D*_*t*_, quantifies microcrack initiation in frozen soil resulting from elevated unfrozen water content under temperature rise. This process weakens cryo-cementation bonds, with its expression defined via the initial elastic modulus as:

9 $$ {\it\text{D}}_{t} = 1 - {{E_{t} } \mathord{\left/ {\vphantom {{E_{t} } {E_{0} }}} \right. \kern-0pt} {E_{0} }} = 1 - \eta$$

where *E*_*0*_ represents the initial elastic modulus of the frozen soil before heating, and *E*_*t*_ denotes the initial elastic modulus of the frozen soil when heated to t℃, and *η* denotes the damage parameter correlated with both the initial modulus and temperature.

The second component, the load-induced damage variable *D*_*p*_, characterizes structural degradation in frozen silty clay under loading. This process initiates with stress concentration at interparticle contacts, propagating microcracks that ultimately compromise soil fabric integrity. Defined as the ratio of failed microscopic units to total units within the soil matrix, its expression is:

10 $$\it {\text{D}}_{p} = {{N_{f} } \mathord{\left/ {\vphantom {{N_{f} } N}} \right. \kern-0pt} N}$$

where *N* represents total number of micro-units within the soil; *N*_*f*_ is number of damaged micro-units within the soil under load.

Assuming the probability of micro-unit damage in frozen silty clay is *f(x)*, the number of damaged micro-units within an arbitrary stress interval [*F*, *F* + *dF*] is *Nf*(*x*)*dx*. As the load increases to *F*, the number of damaged micro-units is given by:

11 $$\it {\text{N}}_{f} \left( {\text{F}} \right) = \int\limits_{0}^{\text{F}} {{\text{Nf}} \left( {\text{x}} \right){\text{d}} {\text{x}} }$$

where *F* represents the unit stress, and *f*(*x*) is the probability distribution function of damage in frozen silty clay.

Substituting Eq. (11) into Eq. (12) yields the damage in frozen silty clay resulting from the applied load:

12 $$\it {\text{D}}_{p} = {{N_{f} } \mathord{\left/ {\vphantom {{N_{f} } N}} \right. \kern-0pt} N} = {{\left[ {\int\limits_{0}^{\text{F}} {{\text{Nf}} \left( {\text{x}} \right){\text{d}} {\text{x}} } } \right]} \mathord{\left/ {\vphantom {{\left[ {\int\limits_{0}^{\text{F}} {{\text{Nf}} \left( {\text{x}} \right){\text{d}} {\text{x}} } } \right]} N}} \right. \kern-0pt} N} = \int\limits_{0}^{\text{F}} {f\left( {\text{x}} \right){\text{d}} {\text{x}} }$$

When selecting a statistical distribution function to characterize the microstructural damage units in frozen soil under loading, several candidate distributions were evaluated: The symmetric assumption of normal distribution^46^ contradicts the unilateral cumulative nature of frozen soil damage; log-normal distribution’s parameters lack the physical interpretability of Weibull distribution^47^; and power-law distribution^48^ fails to provide explicit threshold parameters for correlating with actual soil strength. In contrast, Weibull distribution not only matches the unilateral cumulative damage characteristics of frozen soils but also offers clearly interpretable physical parameters. Its established application in modeling brittle materials like rock and concrete further supports its adoption^49,50^. These comparative advantages justify the selection of Weibull distribution function to describe the statistical distribution of damage units in loaded frozen soil.

Assuming that the distribution of damaged micro-units in frozen silty clay under load adheres to a Weibull distribution^51–53^, the expression for its distribution function is as follows:

13 $$ {\it\text{f}} \left( \varepsilon \right) = \left( {{m \mathord{\left/ {\vphantom {m {F_{0} }}} \right. \kern-0pt} {F_{0} }}} \right)\left( {{\varepsilon \mathord{\left/ {\vphantom {\varepsilon {F_{0} }}} \right. \kern-0pt} {F_{0} }}} \right)^{m - 1} exp\left[ { - \left( {{\varepsilon \mathord{\left/ {\vphantom {\varepsilon {F_{0} }}} \right. \kern-0pt} {F_{0} }}} \right)^{m} } \right]$$

where *F*_*0*_ represents the scaling parameter, which governs the critical strain in the frozen soil damage model and reflects the structural strength of frozen soil; and *m* denotes the shape parameter, controlling the curve morphology in the damage model and characterizing the distribution type of defects in frozen soil.

Substituting Eq. (13) into Eq. (12) yields the damage variable of frozen silty clay resulting from the applied load:

14 $${\it \text{D}}_{p} = \int\limits_{0}^{\it \text{F}} {f\left( {\it \text{x}} \right){\it \text{d}} {\it \text{x}} } = 1 - exp\left[ { - \left( {{\varepsilon \mathord{\left/ {\vphantom {\varepsilon {F_{0} }}} \right. \kern-0pt} {F_{0} }}} \right)^{m} } \right]$$

By combining Eqs. (7, 9 and 14), the total damage variable of frozen silty clay under uniaxial compression during the heating process can be determined as follows:

15 $${\it \text{D}} = 1 - \left( {1 - {\it \text{D}}_{t} } \right)\left( {1 - {\it \text{D}}_{p} } \right) = 1 - \eta exp\left[ { - \left( {{\varepsilon \mathord{\left/ {\vphantom {\varepsilon {F_{0} }}} \right. \kern-0pt} {F_{0} }}} \right)^{m} } \right]$$

#### Parameters of damage model

By simultaneously solving Eqs. (8) and (15), the following relationship is obtained:

16 $$\sigma = {\it \text{E}} \varepsilon \left\{ {\eta exp\left[ { - \left( {{\varepsilon \mathord{\left/ {\vphantom {\varepsilon {F_{0} }}} \right. \kern-0pt} {F_{0} }}} \right)^{m} } \right]} \right\}$$

**Fig. 18:**
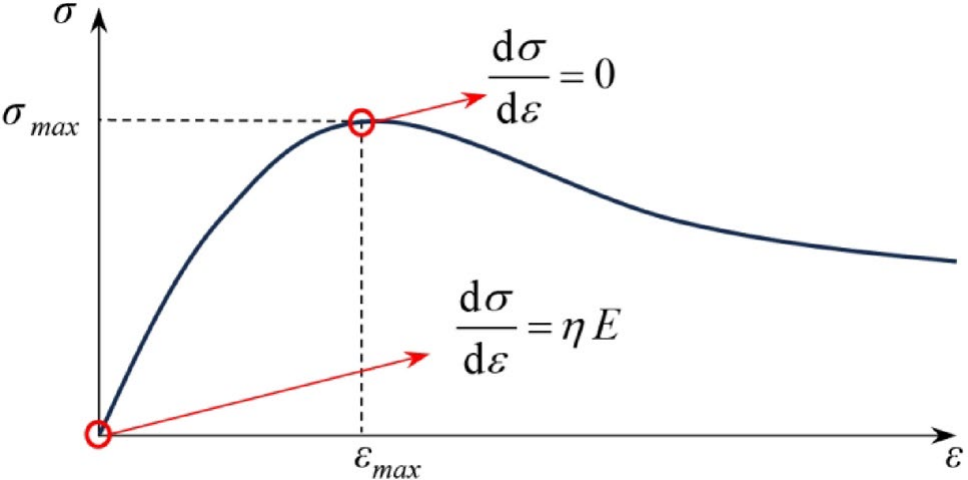
Damage boundary conditions.

As illustrated in Fig. 18, the stress–strain relationship described by Eq. (16) must satisfy the following boundary conditions:

1. When *ε* = 0, *σ* = 0, and $${{d\sigma } \mathord{\left/ {\vphantom {{d\sigma } {d\varepsilon }}} \right. \kern-0pt} {d\varepsilon }} = \eta {\it \text{E}}$$;
2. When $$\varepsilon = \varepsilon_{\max }$$, $$\sigma = \sigma_{\max }$$, and $${{d\sigma } \mathord{\left/ {\vphantom {{d\sigma } {d\varepsilon }}} \right. \kern-0pt} {d\varepsilon }} = 0$$.

The differentiation of Eq. (16) yields:

17 $${{d\sigma } \mathord{\left/ {\vphantom {{d\sigma } {d\varepsilon }}} \right. \kern-0pt} {d\varepsilon }} = \eta {\it \text{E}} \left[ {1 - m\left( {{\varepsilon \mathord{\left/ {\vphantom {\varepsilon {F_{0} }}} \right. \kern-0pt} {F_{0} }}} \right)^{m} } \right]exp\left[ { - \left( {{\varepsilon \mathord{\left/ {\vphantom {\varepsilon {F_{0} }}} \right. \kern-0pt} {F_{0} }}} \right)^{m} } \right]$$

Equations (16) and (17) automatically satisfy Condition (1); substituting Condition (2) into Eqs. (16) and (17) yields:

18 $${\it \text{m}} = - \left\{ {ln\left[ {{{\sigma_{\max } } \mathord{\left/ {\vphantom {{\sigma_{\max } } {\left( {\eta {\text{E}} \varepsilon_{\max } } \right)}}} \right. \kern-0pt} {\left( {\eta {\it \text{E}} \varepsilon_{\max } } \right)}}} \right]} \right\}^{ - 1}$$

19 $${\it \text{F}}_{0} = \varepsilon_{\max } \sqrt[{\it \text{m}} ]{\it \text{m}}$$

Substituting Eqs. (18) and (19) into Eq. (16) and rearranging results in the damage evolution model equation for frozen silty clay under heating and loading conditions.

Substituting the experimental data into Eqs. (18) and (19) yields the various damage model parameters for the frozen silty clay from the Daxing’anling (Table 1).

**Table 1:** Damage model parameters.

Temp (℃)	− 0.5	− 1.0	− 2.0	− 3.0	− 5.0	− 7.5
*m*	0.188062	0.195285	0.201222	0.158913	0.162178	0.166065
*F*_*0*_	0.002086	0.003482	0.005183	0.000146	0.00021	0.00022

#### Validation of damage model

**Fig. 19:**
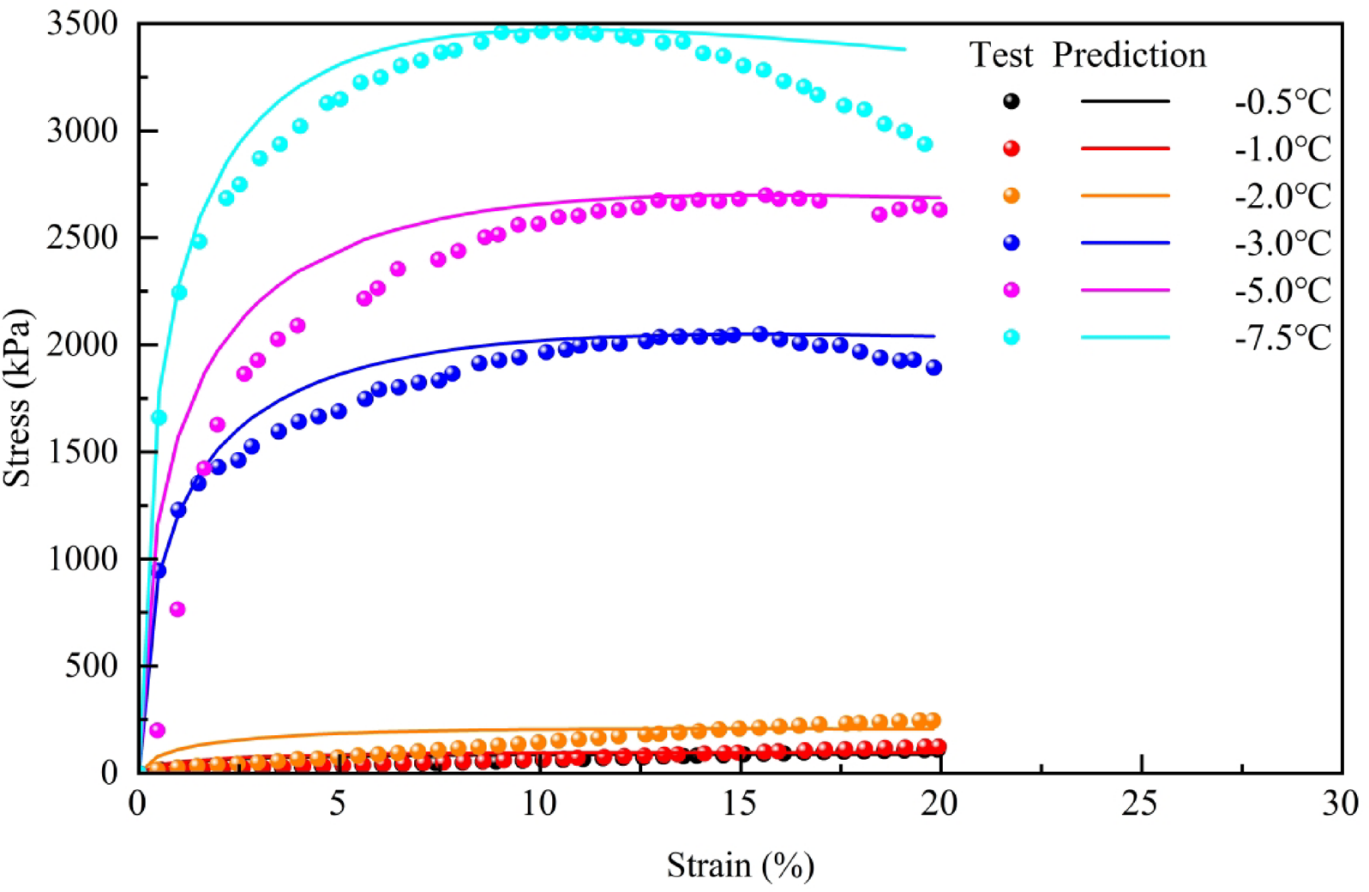
Comparison of test value and prediction value.

Substituting the damage model parameters *m* and *F*_*0*_ for silty clay in the Xing’an Baikal permafrost region at various freezing temperatures from Table 1 into Eq. (16) yields the simulated stress–strain curve for frozen silty clay. The stress–strain curves obtained from both the damage model simulations and experimental tests were plotted together (Fig. 19), with quantitative model accuracy evaluation parameters provided (Table 2) to assess the validity of the damage model for frozen silty clay in the Xing’an-Baikal permafrost region.

**Table 2:** Damage model accuracy assessment.

Temp (℃)	− 0.5	− 1.0	− 2.0	− 3.0	− 5.0	− 7.5
*RMSE*	27.67	32.94	69.80	92.00	261.70	168.49
*MAE*	23.88	28.45	58.19	74.77	141.20	128.96
*R* ^2^	0.14	0.13	0.11	0.94	0.85	0.92

As illustrated in Fig. 19 and Table 2, the stress–strain curves of frozen silty clay from the Xing’an Baikal permafrost region, obtained from experiments, aligns well with the stress–strain curves derived from the damage model. The fit for frozen silty clay at higher temperatures is lower than that for frozen silty clay at lower temperatures prior to reaching failure stress, indicating that elevated soil temperatures under negative conditions enhance the initial damage to the soil. A comparison of the measured and simulated values of failure stress for frozen silty clay reveals that the model accurately predicts the failure stress of this material. Consequently, the damage model proposed in this chapter, which accounts for the warming process of frozen soil, can accurately describe the stress–strain changes of frozen silty clay under heating and cooling conditions in the Xing’an Baikal permafrost region. This offers a reliable theoretical foundation for engineering applications in the region.

#### Advantages and limitations of the model

This study proposes a temperature-load dual-damage coupling model that achieves precise characterization of damage across the entire temperature domain of frozen soil by quantifying the synergistic effects of thermal damage (ice cementation degradation) and mechanical damage (crack propagation). The model effectively reveals the distinct failure mechanisms of both low-temperature and high-temperature frozen soils.

While the proposed damage model effectively characterizes the mechanical behavior of frozen silty clay in the Xing’an Baikal permafrost region, several limitations warrant consideration: First, the model validation was conducted only within the temperature range of − 7.5 to − 0.5 °C, and its predictive accuracy near 0 °C may be compromised due to dramatic unfrozen water content variations. Second, while optimized for frozen silty clay, the model’s applicability to other frozen soil types—such as gravel-rich permafrost or organic-rich frozen soils—with potentially different damage mechanisms requires further investigation. Third, the model was developed based on saturated frozen soil behavior and consequently does not account for the damage characteristics of unsaturated frozen soils. These limitations highlight important directions for future model refinement and extension.

The temperature-load dual-damage model developed in this study has been successfully validated for Xing’an-Baikal permafrost, demonstrating significant potential for future extensions: (1) incorporation of a fatigue damage factor to characterize freeze–thaw cycle effects, enabling application to seasonally frozen soils and active layers; (2) coupling with matric suction damage mechanisms to extend the model’s capability to unsaturated frozen soils.

The damage model proposed in this study demonstrates broad applicability across thermal regimes, being equally valid for both cold permafrost in polar regions and warm permafrost on the Qinghai-Tibet Plateau due to its explicit incorporation of both high-temperature and low-temperature frozen soil behavior. Furthermore, while specifically developed for silty clay, the model can be extended to gravelly frozen soils in Alaska through the inclusion of a grain-size distribution correction coefficient.

## Conclusions

This study investigates the mechanical behavior of frozen silty clay in the Xing’an Baikal permafrost region through uniaxial compression tests and damage modeling, yielding some key findings:

1. The mechanical strength of frozen silty clay demonstrates a temperature-dependent tri-phasic evolution: Between − 0.5 and − 2.0 °C, strength increases gradually (initial tangent modulus: 0.61 MPa/°C; compressive strength: 79.99 kPa/°C) primarily due to enhanced unfrozen water viscosity; From − 2.0 to − 3.0 °C, strength surges dramatically (69.58 MPa/°C and 1842.00 kPa/°C, respectively) as ice cementation becomes dominant, constituting a critical transition zone that warrants intensified foundation monitoring to prevent instability from temperature fluctuations; and below − 3.0 °C, strength growth stabilizes (13.32 MPa/°C and 316.20 kPa/°C) as cementation effects reach saturation. This distinct phased behavior underscores the necessity of temperature-specific considerations in permafrost engineering design and maintenance.
2. The damage model developed by integrating Lemaitre’s strain equivalence principle with Weibull distribution provides accurate predictions of the stress–strain behavior of frozen soils. This modeling framework serves as an effective engineering tool for determining failure thresholds in permafrost regions, enabling optimization of temperature control standards during construction in cold environments.
3. When temperatures drop below − 3.0 °C, the frozen soil undergoes a distinct failure mode transition from plastic to brittle behavior, exhibiting sudden failure under loading conditions. This mechanical transition necessitates enhanced foundation ductility in extreme low-temperature regions to mitigate catastrophic collapse risks.

While the current study has established a damage model specifically tailored for Xing’an Baikal permafrost, several critical research directions merit further investigation: (1) Development of a universal frozen soil damage model to overcome existing limitations and establish a generalized framework for permafrost mechanics research; and (2) Incorporation of freeze–thaw cycle damage factors to evaluate long-term durability under climate-induced thermal fluctuations. These advancements would significantly enhance the model’s applicability across diverse permafrost environments and improve predictive capabilities under changing climatic conditions.

## Data Availability

The datasets generated during and/or analysed during the current study are available from the corresponding author on reasonable request.
